# An ancient Pygo-dependent Wnt enhanceosome integrated by Chip/LDB-SSDP

**DOI:** 10.7554/eLife.09073

**Published:** 2015-08-27

**Authors:** Marc Fiedler, Michael Graeb, Juliusz Mieszczanek, Trevor J Rutherford, Christopher M Johnson, Mariann Bienz

**Affiliations:** 1 MRC Laboratory of Molecular Biology, Francis Crick Avenue, Cambridge Biomedical Campus, Cambridge, United Kingdom; Stowers Institute for Medical Research, United States

**Keywords:** LIM domain-binding protein, beta-catenin, groucho/TLE, RUNX, Osa/ARID1, Wnt-responsive transcription, *D. melanogaster*, human

## Abstract

TCF/LEF factors are ancient context-dependent enhancer-binding proteins that are activated by β-catenin following Wnt signaling. They control embryonic development and adult stem cell compartments, and their dysregulation often causes cancer. β-catenin-dependent transcription relies on the NPF motif of Pygo proteins. Here, we use a proteomics approach to discover the Chip/LDB-SSDP (ChiLS) complex as the ligand specifically binding to NPF. ChiLS also recognizes NPF motifs in other nuclear factors including Runt/RUNX2 and *Drosophila* ARID1, and binds to Groucho/TLE. Studies of Wnt-responsive dTCF enhancers in the *Drosophila* embryonic midgut indicate how these factors interact to form the Wnt enhanceosome, primed for Wnt responses by Pygo. Together with previous evidence, our study indicates that ChiLS confers context-dependence on TCF/LEF by integrating multiple inputs from lineage and signal-responsive factors, including enhanceosome switch-off by Notch. Its pivotal function in embryos and stem cells explain why its integrity is crucial in the avoidance of cancer.

**DOI:** http://dx.doi.org/10.7554/eLife.09073.001

## eLife digest

In animals, cells have to be able to communicate with neighboring cells in order to generate and maintain the different tissues and organs. One ancient method of cell communication that is used in all animals is the Wnt signaling pathway. In this pathway, a cell secretes a protein called Wnt, which binds to a Wnt receptor present on the surface of another cell. This triggers a cascade of signals inside the second cell that leads to the activation of proteins called TCF factors. These proteins bind to regions of DNA called enhancers to trigger the expression of particular genes that control the development of the animal.

Hyperactive Wnt signaling in humans can result in cancer, so Wnt signaling is tightly controlled to avoid this. One of the proteins that regulates Wnt signaling is called Groucho and it interacts with TCF to prevent it from activating genes in the absence of a Wnt signal. However, when Wnt is present, a protein called Pygo overcomes this repression by Groucho to activate TCF, but it is not clear how this works. Fiedler, Graeb, Mieszczanek et al. discovered that Pygo directly binds to a protein complex called Chip/LDB-SSDP (or ChiLS for short). ChiLS is able to associate with TCF enhancers through its association with Groucho.

Fiedler, Graeb, Mieszczanek et al. observed that ChiLS can also interact with a number of other proteins that control body formation. This enables ChiLS to integrate multiple signals that regulate the activity of TCF factors. Fiedler, Graeb, Mieszczanek et al. named this complex the ‘Wnt enhanceosome’ because it serves to activate the expression of genes in response to Wnt signaling.

Fiedler, Graeb, Mieszczanek et al. analyzed the role of the Wnt enhanceosome during the development of the fly wing and the embryo's midgut. Many genes that are required to form these organs were switched on by the Wnt enhanceosome. This study shows that ChiLS and Pygo are core components of a large complex of proteins that regulate animal development. The next challenge is to study how the components of this complex work together to regulate the enhancers in response to different signals.

**DOI:** http://dx.doi.org/10.7554/eLife.09073.002

## Introduction

TCF/LEF factors (TCFs) were discovered as context-dependent architectural factors without intrinsic transactivation potential that bind to the T cell receptor α (TCRα) enhancer via their high mobility group (HMG) domain (Waterman and Jones, 1990; Giese et al., 1992). They facilitate complex assemblies with other nearby enhancer-binding proteins, including the signal-responsive CRE-binding factor (CREB) and the lineage-specific RUNX1 (also called Acute Myeloid Leukemia 1, AML1). Their activity further depends on β-catenin, a transcriptional co-factor activated by Wnt signaling, an ancient signaling pathway that controls animal development and stem cell compartments, and whose dysregulation often causes cancer (Clevers, 2006). The context-dependence of TCFs is also apparent in other systems, for example in the embryonic midgut of *Drosophila* where dTCF integrates multiple signaling inputs with lineage-specific cues during endoderm induction (Riese et al., 1997). The molecular basis for this context-dependence remains unexplained.

In the absence of signaling, T cell factors (TCFs) are bound by the Groucho/Transducin-like Enhancer-of-split (Groucho/TLE) proteins, a family of co-repressors that silence TCF enhancers by recruiting histone deacetylases (HDACs) (Turki-Judeh and Courey, 2012) and by ‘blanketing’ them with inactive chromatin (Sekiya and Zaret, 2007). TLEs are displaced from TCFs by β-catenin following Wnt signaling, however this is not achieved by competitive binding (Chodaparambil et al., 2014) but depends on other factors. One of these is Pygopus (Pygo), a conserved nuclear Wnt signaling factor that recruits Armadillo (*Drosophila* β-catenin) via the Legless/BCL9 adaptor to promote TCF-dependent transcription (Kramps et al., 2002; Parker et al., 2002; Thompson et al., 2002). Intriguingly, Pygo is largely dispensable in the absence of Groucho (Mieszczanek et al., 2008), which implicates this protein in alleviating Groucho-dependent repression of Wg targets.

**Figure 1.**
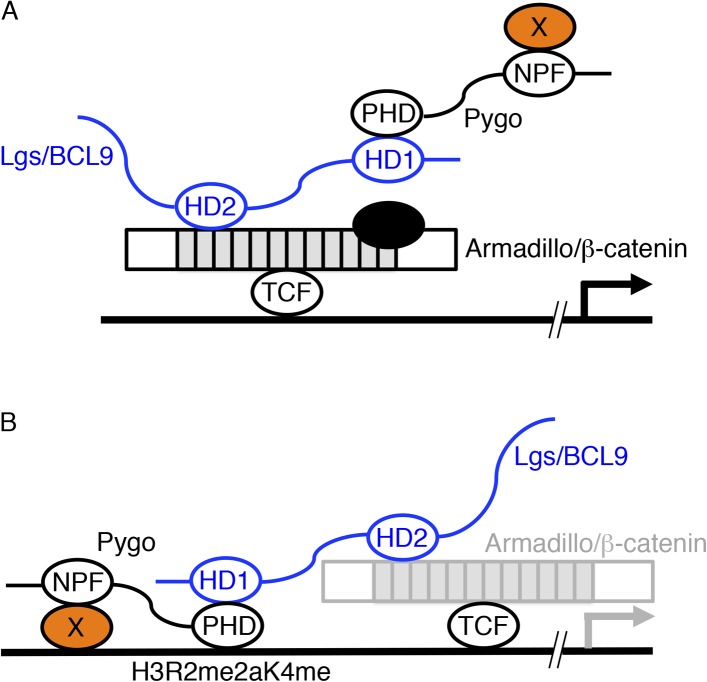
Two models of Pygo function. (**A**) The co-activator model (Kramps et al., 2002; Hoffmans et al., 2005): the NPF ligand (X, orange) is a transcriptional co-activator recruited to dTCF enhancers *exclusively* during Wnt signaling through the Pygo-Legless/BCL9 adaptor chain (Stadeli and Basler, 2005), co-operating with other transcriptional co-activators recruited to the C-terminus of Armadillo (such as chromatin remodelers and modifiers, black) in stimulating Wg-induced transcription. (**B**) The Armadillo-loading model (Townsley et al., 2004): the NPF ligand (X, orange) mediates constitutive tethering of Pygo to dTCF enhancers *prior* to Wg signaling, jointly with PHD-mediated recognition of H3K4me1 (marking poised enhancers; Kharchenko et al., 2011) or H3R2me2aK4me1 (marking silenced enhancers in the process of being activated; Kirmizis et al., 2007), priming these enhancers for Wg responses via its ability to capture Armadillo (once available during Wg signaling, indicated by grey) through the Legless/BCL9 adaptor. In both models, the homology domain 1 (HD1) of Lgs/BCL9 binds to the Pygo PHD finger, while HD2 binds to the N-terminus of the Armadillo Repeat Domain (light grey) of Armadillo/β-catenin (Kramps et al., 2002; Sampietro et al., 2006; Fiedler et al., 2008; Miller et al., 2013). **DOI:** http://dx.doi.org/10.7554/eLife.09073.003

Pygo has a C-terminal plant homology domain (PHD) and an N-terminal asparagine proline phenylalanine (NPF) motif, each essential for development and tissue patterning (Mosimann et al., 2009). Much is known about the PHD finger, which binds to Legless/BCL9 (Kramps et al., 2002) and to histone H3 tail methylated at lysine 4 via opposite surfaces (Fiedler et al., 2008; Miller et al., 2013) that are connected by allosteric communication (Miller et al., 2010). By contrast, the NPF ligand is unknown, but two contrasting models have been proposed for its function (Figure 1).

Here, we use a proteomics approach to discover that the NPF ligand is an ancient protein complex composed of Chip/LDB ((Lin-11 Isl-1 Mec-3-) LIM-domain-binding protein) and single-stranded DNA-binding protein (SSDP), also called SSBP. This complex controls remote Wnt- and Notch-responsive enhancers of homeobox genes in flies (Bronstein and Segal, 2011), and remote enhancers of globin and other erythroid genes in mammals, integrating lineage-specific inputs from LIM-homeobox (LHX) proteins and other enhancer-binding proteins (Love et al., 2014). Using nuclear magnetic resonance (NMR) spectroscopy, we demonstrate that Chip/LDB-SSDP (ChiLS) binds directly and specifically to Pygo NPFs, and also to NPF motifs in Runt-related transcription factors (RUNX) proteins and Osa (*Drosophila* ARID1), whose relevance is shown by functional analysis of *Drosophila* midgut enhancers. Furthermore, we identify Groucho as another new ligand of ChiLS by mass spectroscopy. We thus define the core components of a Wnt enhanceosome assembled at TCF enhancers via Groucho/TLE and RUNX, primed for timely Wnt responses by ChiLS-associated Pygo. The pivotal role of ChiLS in integrating the Wnt enhanceosome provides a molecular explanation for the context-dependence of TCFs.

## Results

### ChiLS is the ligand for Pygo NPF

**Figure 2.**
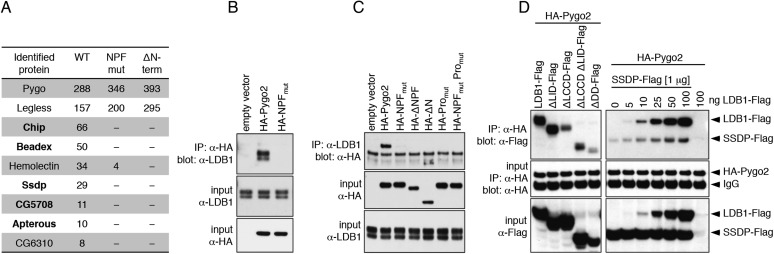
Pygo NPF binds to ChiLS. (**A**) Top proteins associated with wt but not NPF-mutant Pygo in S2 cells (unweighted spectral counts >95% probability are given); *bold*, ChiLS and its ligands. (**B**–**D**) Western blots of coIPs from transfected HEK293T cells, showing NPF-dependent coIP of (**B, C**) endogenous LDB1 with HA-Pygo2 or (**D**) wt vs truncated LDB1-Flag with HA-Pygo2 (*left*), and LDB1-Flag +/− SSDP-Flag with HA-Pygo2 (*right*; increasing amounts of LDB1-Flag indicated above panel). **DOI:** http://dx.doi.org/10.7554/eLife.09073.004

To identify the NPF ligand of *Drosophila* Pygo, we inserted various tags into its low-complexity linker that separates NPF from PHD (Figure 2—figure supplement 1A), and used stably transfected S2 cell lines expressing wild-type (wt) or NPF-mutant versions, for tandem-affinity purification of associated proteins and identification by mass spectrometry. We thus discovered Chip, SSDP and three LIM domain proteins—Beadex and CG5708 (both LIM-only proteins, LMO) and Apterous (an LHX)—amongst the top hits specifically associated with wt but not mutant Pygo (Figure 2A). These proteins are known to form a complex: Chip dimerizes through DD (dimerization domain) and binds to SSDP through LDB/Chip conserved domain (LCCD) and to LIM domains through LIM-interacting domain (LID). The latter allow ChiLS to associate with enhancers, either directly through LHX (e.g., Apterous), or indirectly through LMO adaptors that bind to bHLH (e.g., Achaete/Scute) and GATA factors (e.g., Pannier) (Bronstein and Segal, 2011; Love et al., 2014). Indeed, LMOs displace LHXs from ChiLS by virtue of their high expression level and/or high affinity for LID (Milan and Cohen, 1999; Ramain et al., 2000; Matthews et al., 2008), and are thus capable of switching from LHX to GATA/bHLH. We also found ChiLS components associated with Pygo2 in stably transfected HEK293T cell lines, and with recombinant triple-NPF baits in lysates from mouse brains and colorectal cancer cell lines (Figure 2—figure supplement 1B–D).

**Figure 2—figure supplement 1.**
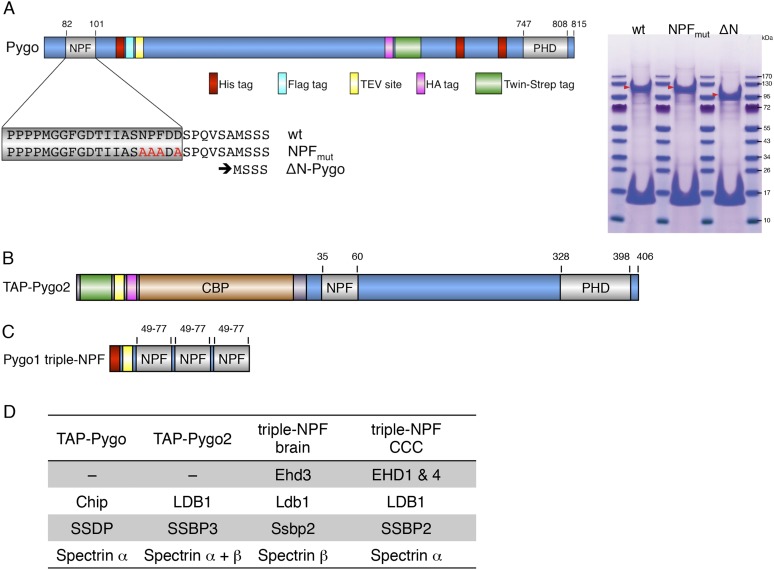
Baits used for mass spectrometry analysis. (**A**) Cartoon of tandem-tagged *Drosophila* Pygo bait, generated for expression in stably transfected S2 cells (continuously selecting with 5 μg ml^−1^ puromycin), and subsequent tandem purification (TAP) of associated proteins using α-Flag followed by Strep-tactin pull-down; red arrowheads indicate wt and mutant Pygo baits after electrophoresis on a stained polyacrylamide gel prior to excision for mass spectrometry analysis. (**B**) Cartoon of tandem-tagged human Pygo2 bait (subcloned in V51 pGLUE), for expression in stably transfected HEK293T cells (continuously selecting with 2 μg ml^−1^ puromycin), for TAP purification of associated proteins with streptavidin followed by Calmodulin resin, essentially adopting a protocol previously described (Angers et al., 2006). (**C**) Cartoon of triple-NPF bait from mouse Pygo1, generated by trimerizing an extended NPF motif (amino acids 49-77), or a corresponding NPF > AAA triple mutant, for Ni-NTA pull-down experiments with lysates from fresh mouse brains (strain C57Bl6J), or from SW480 or COLO320 colorectal cancer cell (CCC) lines (obtained from Marc de la Roche; de la Roche et al., 2014). Interacting proteins were eluted with 6M urea, and samples were prepared for analysis using a filter-aided sample preparation method, essentially as described (Wisniewski et al., 2009). (**D**) Hits identified with TAP-Pygo and TAP-Pygo2 baits in both S2 and HEK293T cells, respectively (see **A**, **B**), and overlap with hits identified by triple-NPF immunoprecipitations from mouse brain and CCC lysates (see **C**), which include ChiLS components. Note that EHD paralogs were only found in the lysate-based approaches (amongst the top hits, due to the cytoplasmic abundance of these proteins) but not with full-length Pygo or Pygo2; spectrins were also commonly found, but the significance of this is unclear. **DOI:** http://dx.doi.org/10.7554/eLife.09073.005

Co-immunoprecipitation (coIP) assays in transfected HEK293T cells revealed that only Chip but none of the other hits coIPed with Pygo. Furthermore, wt but not mutant HA-Pygo2 coIPs with endogenous LDB1, and vice versa (Figure 2B,C). The conserved proline cluster upstream of NPF is also required for binding, consistent with transcription assays in S2 cells that indicated the function of this cluster (Stadeli and Basler, 2005).

Testing truncations of LDB1 for coIP with Pygo2, we found that LID is dispensable for binding, whereas dimerization seems important since there is little interaction with Pygo2 in the absence of DD (Figure 2D). Importantly, LDB1 coIPs with Pygo2 in a dose-dependent way upon co-expression, whereas SSDP hardly coIPs with Pygo2 in the absence of exogenous LDB1, even at high SSDP excess (Figure 2D). The reverse could not be established since LDB1 overexpressed on its own is highly unstable (Figure 2D), being targeted for proteasomal degradation in the absence of SSDP (Xu et al., 2007b). This reinforces the notion that Pygo NPF binds to Chip/LDB.

### A dimer-tetramer architecture of ChiLS

**Figure 3.**
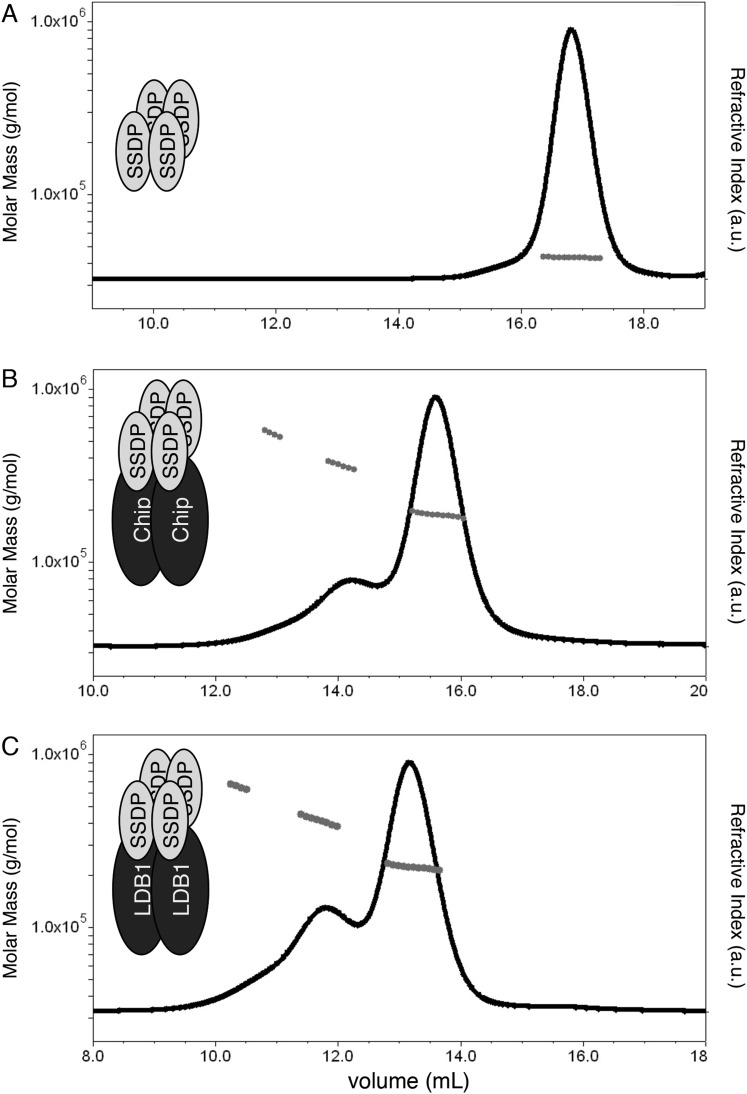
Stoichiometry of the ChiLS complex. SEC-MALS of (**A**) Lip-SSDP_1-92_, or co-expressed (**B**) Lip-SSDP_1-92_ + MBP-Chip_205-436_ or (**C**) Lip-SSDP_1-92_ + MBP-LDB1_56-285_; solid black lines, elution profile as detected by the RI detector; grey circles, molecular mass; cartoons in panels indicate stoichiometries consistent with the measured molecular masses. **DOI:** http://dx.doi.org/10.7554/eLife.09073.006

To test direct binding of NPF to ChiLS, we purified the NPF-interacting DD-LCCD fragments of Chip and LDB1 after bacterial expression. Both have a strong tendency to aggregate if expressed on their own, but become soluble if co-expressed with SSDP_1-92_ (i.e., the LisH domain-containing N-terminus of the fly protein, without its unstructured tail; this LisH domain is nearly identical to its human counterpart, with two residues only semi-conserved). Expressed by itself, SSDP_1-92_ is soluble and elutes as a single peak after gel filtration, regardless of concentration (Figure 3A). Size exclusion chromatography coupled to multi-angle light scattering (SEC-MALS) revealed an average apparent molecular mass of 43 kDa for the protein in this peak, which corresponds to a tetramer (expected molecular mass 42.7 kDa). Evidently, SSDP_1-92_ forms a stable tetramer in solution. We note that LisH domains typically form dimers, but some tetramerize (e.g., that of TBL1, a subunit of a HDAC-recruiting co-repressor; Oberoi et al., 2011).

**Figure 3—figure supplement 1.**
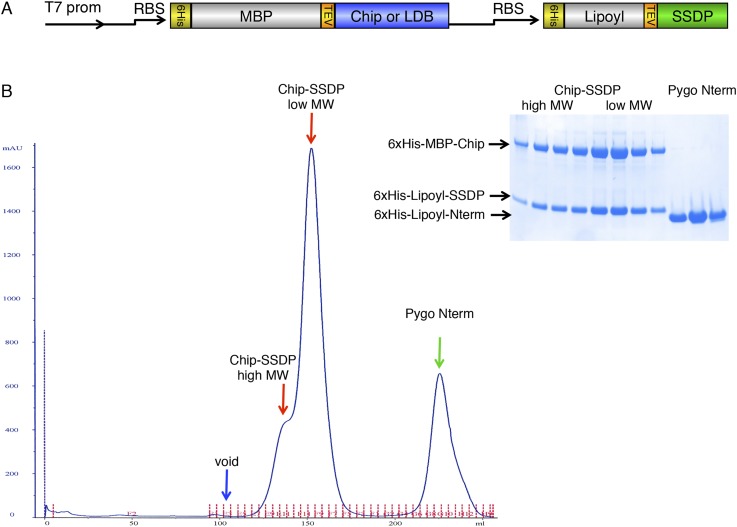
Gel filtration of ChiLS. (**A**) Bi-cistronic expression vector (with T7 promoter) used for bacterial expression of ChiLS complex, encoding 6xHis-MBP-Chip_205-436_ or 6xHis-MBP-LDB1_56-285_ followed by 6xHis-Lipoyl-SSDP_1-92_ with its own ribosome-binding site (RBS). (**B**) Elution profile of MBP-Chip_205-436_, Lipoyl-SSDP_1-92_ (after co-expression with vector shown in **A**) and incubation with excess 6xHis-Lipoyl-Pygo_67-107_ (Pygo Nterm), revealing three main species, as indicated above graph, corresponding to free Pygo Nterm, Chip-SSDP_low_ and Chip-SSDP_high_ complexes, as shown by polyacrylamide gel electrophoresis (inset); note that the high-molecular weight shoulder (Chip-SSDP_high_) corresponds to a dimer of the major ∼190 kDa low-molecular weight species (Chip-SSDP_low_), whereby the latter corresponds to an MBP-Chip dimer bound to an SSDP tetramer (see Figure 3B). Chip-SSDP_low_ and Chip-SSDP_high_ complexes cannot be further separated by subsequent anion exchange chromatography, and do not seem to interconvert, as judged by iterative G-200 gel chromatography. Both complexes bind to ^15^N-Pygo-NPF (see Figure 4), however, Pygo Nterm dissociates from ChiLS during gel filtration (see inset), indicating a low affinity to ChiLS (estimated to be mid-μM). **DOI:** http://dx.doi.org/10.7554/eLife.09073.007

On co-expression, Chip_205-436_ and SSDP_1-92_ form a stable complex that elutes consistently as one main peak with a higher-mass shoulder after gel filtration (Figure 3—figure supplement 1), regardless of concentration. The peaks of these two species are not baseline-resolved during SEC-MALS, but their average masses of ∼190 kDa and ∼380 kDa suggest that the shoulder is a dimer of the major ∼190 kDa species (Figure 3B). The latter thus corresponds to an MBP-Chip dimer bound to an SSDP tetramer (expected 181 kDa), the only possible stoichiometry that fits the observed molecular mass. The same architecture is also found for the human LDB1-SSDP complex (Figure 3C), revising previous models of ChiLS (e.g., Bronstein and Segal, 2011).

### Pygo NPF binds directly to ChiLS

**Figure 4.**
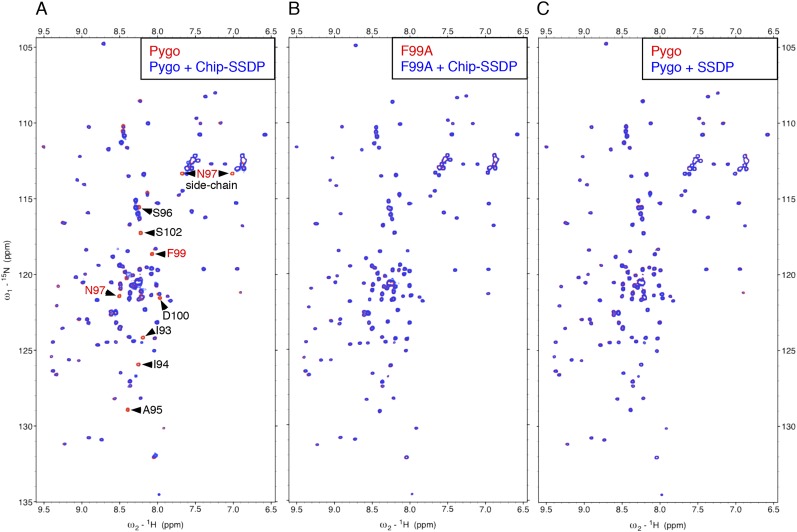
Direct NPF-dependent binding of Pygo by ChiLS. Overlays of HSQC spectra of 50 μM ^15^N-labeled wt or F99A mutant Pygo_67-107_ alone (red) or probed with (**A**, **B**) MBP-Chip_205-436_- Lip-SSDP_1-92_ or (**C**) Lip-SSDP_1-92_ alone (blue); interacting residues are labeled, with NPF in red (binding to P is not detectable by HSQCs). The HSQC obtained with 50 μM of minimal ^15^N-labeled Pygo_87-102_ is indistinguishable from that shown in (**A**). **DOI:** http://dx.doi.org/10.7554/eLife.09073.008

To monitor direct NPF-dependent binding, we incubated purified Chip-SSDP with wt and mutant ^15^N-labeled Lipoyl-tagged (Lip) Pygo_67-107_ (^15^N-Nterm) and recorded heteronuclear single-quantum correlation (HSQC) spectra by NMR. We thus observed clear line broadenings with wt (Figure 4A) but not with F99A mutant ^15^N-Nterm (bearing a point mutation in NPF; Figure 4B). Incubation of wt ^15^N-Nterm with purified SSDP_1-92_ did not produce any spectral changes (Figure 4C), confirming that SSDP does not bind NPF (Figure 2D). We were unable to test binding to Chip_205-436_ alone, owing to its aggregation. LDB1-SSDP also binds to wt but not F78A mutant Pygo2 (Figure 4—figure supplement 1).

**Figure 4—figure supplement 1.**
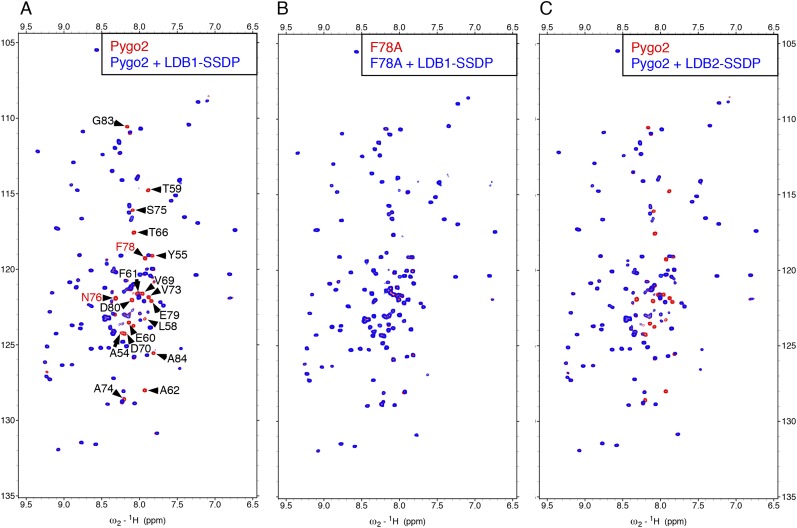
Direct NPF-dependent binding of Pygo2 by ChiLS. Overlays of BEST-TROSY spectra of 50 μM ^15^N-labeled (**A, C**) wt or (**B**) F78A mutant human Pygo2_37-93_ alone (red) or probed with 100 μM (**A, B**) MBP-LDB1_56-285_- Lipoyl-SSDP_1-92_ (blue) or (**C**) MBP-LDB2_20-246_- Lipoyl-SSDP_1-92_ (blue); interacting residues are labeled, with NPF motif in red (binding to P is not detectable by BEST-TROSY). **DOI:** http://dx.doi.org/10.7554/eLife.09073.009

**Figure 5.**
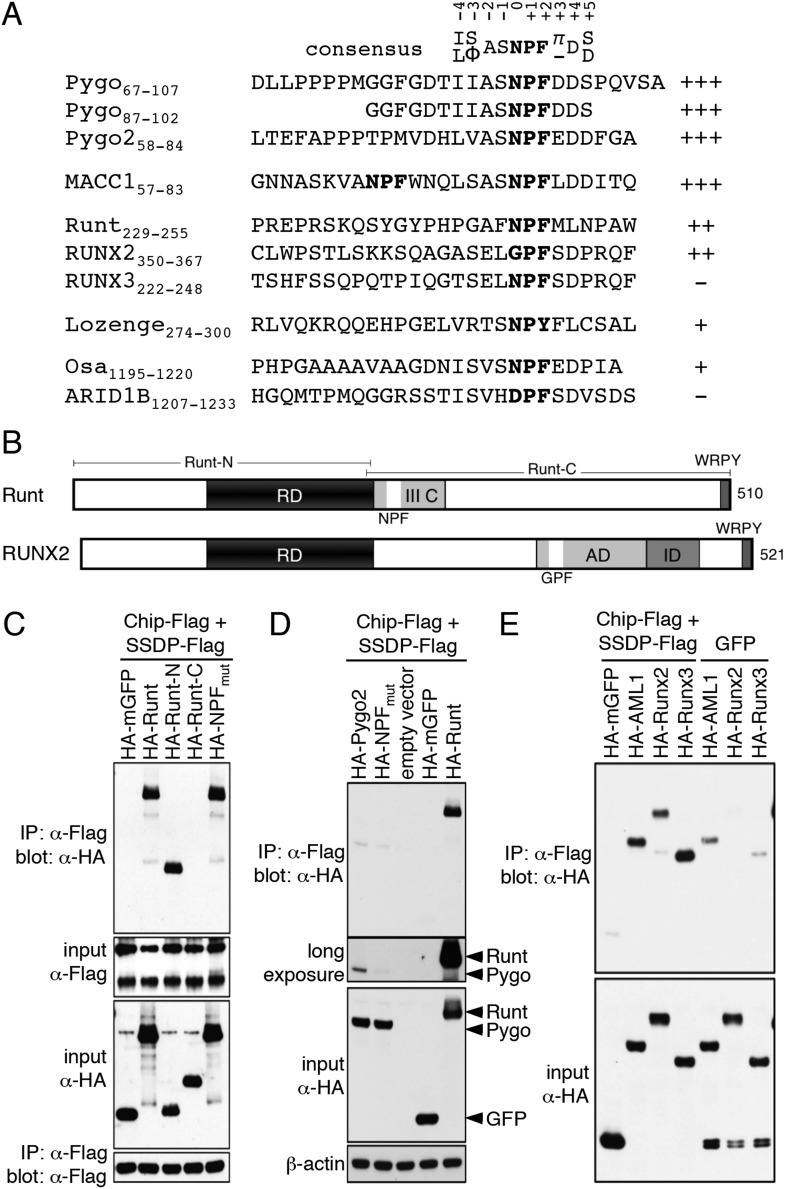
ChiLS binds to RUNX NPFs. (**A**) Summary of NMR binding assays of ^15^N-labeled NPF fragments probed with ChiLS; +–+++, estimates of binding affinities, based on minimal ChiLS concentrations required for line-broadening; −, no binding (see also Figure 4); *top*, preferred NPF context in strong binders (numbering of positions as in de Beer et al., 2000). (**B**) Schematic of RUNX orthologs, with DNA-binding domain (RD, black), region IIIC, activation and inhibitory domains (AD, ID), NPF (or GPF) and WRPY indicated. (**C**–**E**) Western blots as in Figure 2, showing coIP between co-expressed proteins as indicated above panels; for Runt-N and Runt-C, see (**B**); mGFP (control). **DOI:** http://dx.doi.org/10.7554/eLife.09073.010

Assignments of double-labeled protein allowed us to determine that the ChiLS ‘interaction footprints’ span 10 NPF-spanning residues in Pygo, and 25 residues in Pygo2. A minimal 16-mer without the proline cluster produces a comparable interaction (Figure 5A), showing that this cluster is dispensable in this binding assay. Its requirement in cell-based assays may reflect the need for a rigid spacer between NPF and the upstream nuclear localization signal. Notably, the residues immediately flanking the NPF are conserved (Figure 5A) and may contribute to binding (see ‘Discussion’).

**Figure 5—figure supplement 1.**
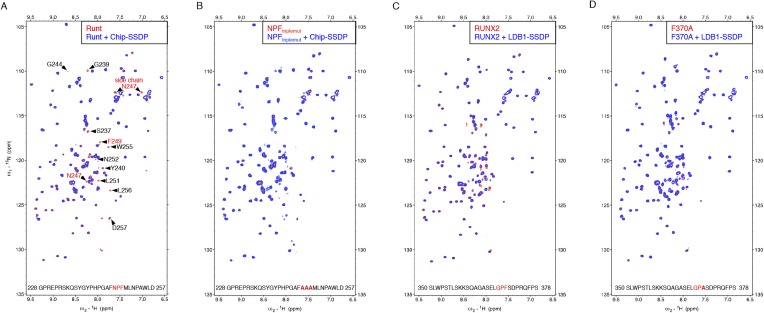
Specific recognition of RUNX NPFs by ChiLS. (**A**, **B**) Overlays of HSQC spectra of 50 μM ^15^N-labeled 6xHis-Lipoyl-tagged (**A**) wt or (**B**) NPF > AAA triple-mutant Runt-NPF_228-257_ alone (red) or probed with 300 μM MBP-Chip_205-436_-Lipoyl-SSDP_1-92_ (blue), as indicated in panels; interacting residues are labeled in (**A**). (**C, D**) Overlays of HSQC spectra of 50 μM ^15^N-labeled 6xHis-Lipoyl-tagged (**C**) wt or (**D**) F > A mutant RUNX2-NPF_350-378_ alone (red) or probed with 300 μM MBP-LDB1_56-285_- Lipoyl-SSDP_1-92_ (blue), as indicated in panels. Note that a triple-NPF mutation (NPF > AAA) is required to block binding of Runt to ChiLS (**B**) while a single F > A mutant Runt still binds to ChiLS (at this concentration). Sequences of ^15^N-labeled NPF peptides are given in the panels (*red*, NPF motifs; *bold*, mutated residues). RUNX3 binding was undetectable with NPF-containing peptides from human RUNX3 (amino acids 227–249; SQPQTPIQGTSEL**NPFSD**PRQFD) or mouse Runx3 (amino acids 214-269). **DOI:** http://dx.doi.org/10.7554/eLife.09073.011

**Figure 5—figure supplement 2.**
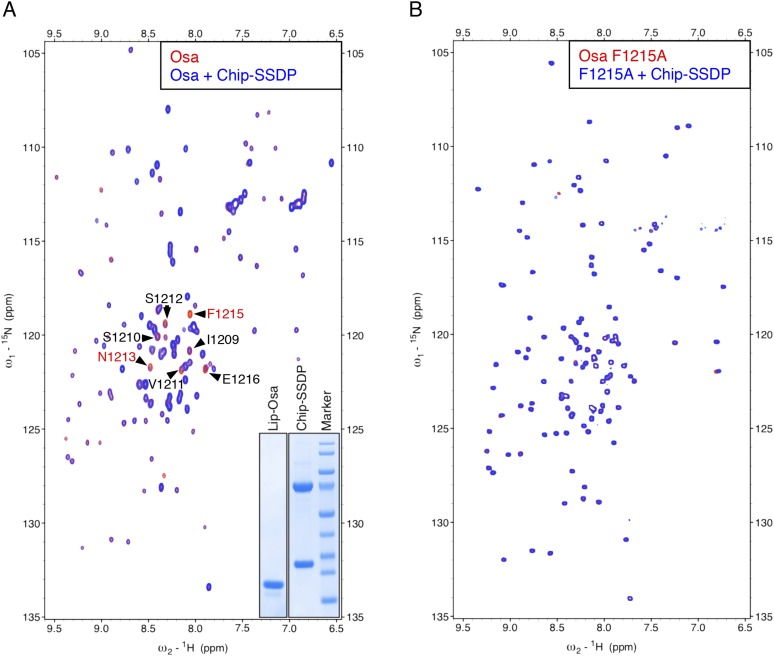
Specific recognition of Osa NPF by ChiLS. Overlays of (**A**) HSQC or (**B**) BEST-TROSY spectra of 50 μM ^15^N-labeled 6xHis-Lipoyl-tagged (**A**) wt or (**B**) F1215A mutant Osa_1195-1221_ alone (red), or probed with 300 μM MBP-Chip_205-436_- Lipoyl-SSDP_1-92_ (blue), as indicated in panels; interacting residues are labeled in (**A**). Inset in (**A**) shows purified 6xHis-Lipoyl-Osa_1195-1220_ and MBP-Chip_205-436_- Lipoyl-SSDP_1-92_ complex separated by polyacrylamide gel electrophoresis. Note also that the NPF-containing peptide we tested for ARID1B was negative, however, this large protein contains further matches to the NPF consensus sequence that might bind ChiLS (to be tested in future studies). The same applies for its ARID1A paralog. **DOI:** http://dx.doi.org/10.7554/eLife.09073.012

**Figure 5—figure supplement 3.**
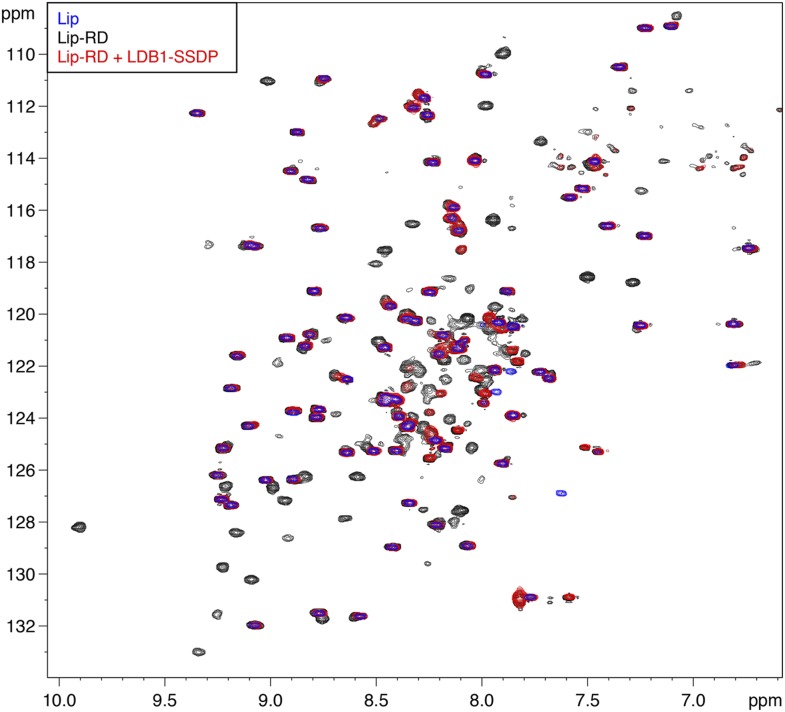
Direct binding of RUNX RD by ChiLS. Overlays of BEST-TROSY spectra of ^15^N-labeled 6xHis-Lipoyl (blue), 6xHis-Lipoyl-RUNX2-RD_102-234_ (black) and 6xHis-Lipoyl-RUNX2-RD_102-234_ + 300 μM MBP-LDB1_56-285_- Lipoyl-SSDP_1-92_ (red), as indicated in panels. Note that the resonances affected by incubation with LDB1-SSDP are predominantly RD-specific (black) whereas most Lip residues (marked by blue) are unaffected. **DOI:** http://dx.doi.org/10.7554/eLife.09073.013

### ChiLS recognizes NPF motifs in RUNX proteins

To identify other NPF-containing proteins as putative ChiLS ligands, we conducted genome-wide database searches for matches to NPFDD-like motifs. We thus found NPFs in several enhancer-binding proteins (Figure 5A), notably in RUNX and Osa/ARID1 (the DNA-binding subunit of SWI/SNF chromatin remodeling complexes; Wu and Roberts, 2013), and also in MACC1 (metastasis-associated in colon cancer 1) whose molecular function is unknown (Stein et al., 2009). NPF-containing fragments from MACC1, RUNX2, Runt and Lozenge (fly RUNX proteins) and Osa tested positive in NMR binding assays, while those from ARID1B and RUNX3 were negative—somewhat curious in the latter case, given that its NPF motif resembles that of Runt (Figure 5A). Testing of NPF mutants confirmed NPF-specific binding (Figure 5—figure supplements 1, 2). Note that ARID1B (and its paralog ARID1A) are large proteins with multiple putative NPFs, whose binding remains to be tested.

Given the linkage between RUNX and TCF in the TCRα enhancer, we decided to pursue this interaction further. In Runt, the NPF is within a short conserved sequence block abutting its Runt domain (the DNA-binding domain; Figure 5B), called region IIIC which is conserved in Runt orthologs of other invertebrates and functionally relevant in *Drosophila* (Walrad et al., 2010). In vertebrate RUNX2/3, the NPFs are further downstream, at the start of a conserved sequence block (Figure 5B). Interestingly, AML1 paralogs do not exhibit an NPF motif at this position, although we note a near-invariant GPFQT/A motif further downstream in all three RUNX paralogs, which could potentially also bind to ChiLS.

CoIP assays revealed that full-length Runt coIPs efficiently with ChiLS (Figure 5C), more so than Pygo (Figure 5D), pointing to a second (stronger) interaction between Runt and ChiLS. Indeed, the N-terminal half of Runt (without NPF) coIPs with ChiLS (and vice versa), as does an NPF-mutant Runt, while the C-terminal half of Runt does not (Figure 5C). Evidently, the affinity of the Runt NPF for ChiLS (estimated to be high μM; Figure 5—figure supplement 1) is too low for Runt to remain associated with ChiLS during coIP. Rather, this interaction depends on the Runt domain to which ChiLS binds directly, as can be shown by NMR (Figure 5—figure supplement 3). Efficient coIP with ChiLS was also observed for AML1, Runx2 and Runx3 (Figure 5E), consistent with the previously reported association of Runx1 with Ldb1 in differentiated mouse erythroleukemic cells (Meier et al., 2006).

### Runt acts through signal-responsive HOX midgut enhancers

**Figure 6.**
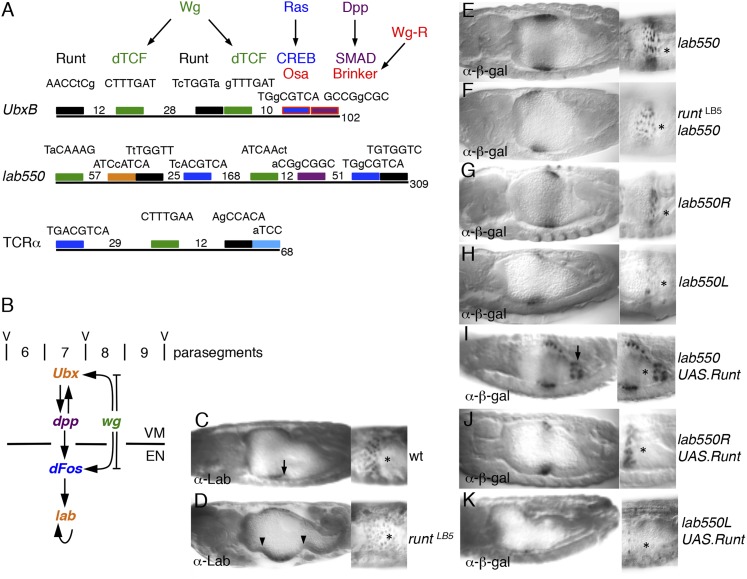
Runt acts through the *labial* midgut enhancer. (**A**) Cartoon of *UbxB*, *lab550* and *TCRα* enhancers, with the following binding sites (responding to signals, as indicated in brackets): *green*, dTCF (Wg); *purple*, SMAD (Dpp); *light-blue*, Ets (Ras); *blue*, CRE (Ras); in *UbxB*, the SMAD binding site also mediates Wg-mediated repression by Brinker (*red*), and CRE mediates Osa-mediated repression (see text); *black*, RUNX; *orange*, Labial; residue numbers between binding sites are given (*right*, total length; note that *UbxB* and *lab550* extend beyond these modules which however contain all known functional binding sites); capital letters, matches to binding site consensus sequences (C/G G C/G G G T C/G for RUNX; Melnikova et al., 1993). (**B**) Cartoon of endoderm induction, color-coded as in (**A**); V, midgut constrictions at parasegment boundaries. (**C, D**) 14 hr old embryos stained with α-Labial; arrow marks incipient second midgut constriction, lacking in *runt* mutants (which only form first and third constrictions, arrowheads). (**E**–**K**) 12–14 hr old embryos bearing wt or mutant *lab550* as indicated on the right, stained with α-β-galactosidase; high magnification views are imaged at different focal planes, to highlight Wg-dependent expression gradients (Wg sources indicated by asterisks). **DOI:** http://dx.doi.org/10.7554/eLife.09073.014

RUNX are context-dependent enhancer-binding proteins that control transcription of master-regulatory genes in *Drosophila* and mammals, co-operating with TCFs (e.g., in the TCRα enhancer; Figure 6A), but also with other signaling inputs including TGF-β/SMAD and Notch (Canon and Banerjee, 2000; Chuang et al., 2013). Indeed, the midgut enhancers from the homeotic (HOX) genes *Ultrabithorax* (*Ubx*) and *labial* each contains two putative RUNX binding sites, linked to functional signal response elements such as dTCF binding sites and CREs (Figure 6A). We exploited these enhancers, to test whether Runt directly controls *Ubx* and *labial* during endoderm induction (Figure 6B). This seemed possible, given that *runt* is required for normal *Ubx* expression in the embryonic visceral mesoderm (Tremml and Bienz, 1989).

**Figure 6—figure supplement 1.**
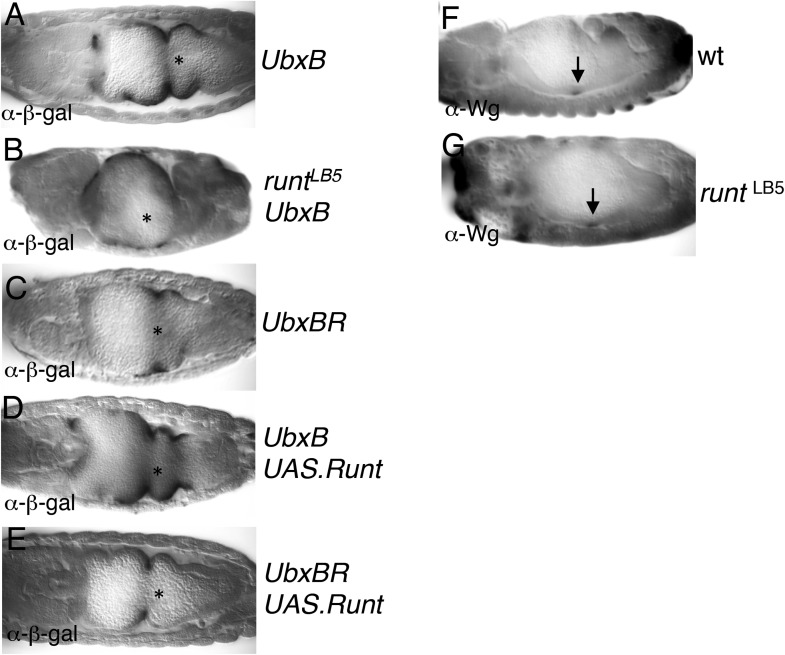
Runt acts through the *Ubx* midgut enhancer. (**A**–**E**) 14–15 hr old wt or *runt* mutant embryos, bearing wt or mutant *UbxB* enhancers (Thuringer et al., 1993), with or without mesodermal GAL4-mediated overexpression of Runt (using *24B.GAL4*) as indicated in panels, fixed and stained with α-β-galactosidase (α-β-gal) antibody; Wg sources (in parasegment 8 of the visceral mesoderm; see Figure 6B) are indicated by asterisks. The expression of the mutant enhancer (*UbxBR*) is reduced, and seen in fewer cells (**C**), similarly to the expression of the wt enhancer in *runt* mutant embryos (**B**), as in the case of *lab550* (Figure 6F,G,J), although in both cases, mutating the Runt binding sites has a somewhat stronger effect on expression than loss of Runt (possibly because of a maternal contribution in the *runt* mutant embryos, which is difficult to rule out). Note also that this minimal enhancer mediates expression posteriorly to parasegment 7 (the *Ubx* expression domain in the visceral mesoderm), partially escaping repression by high Wg signaling levels near the Wg source (Yu et al., 1998), which depends on displacement of SMAD by the Groucho-recruiting Brinker co-repressor (Saller and Bienz, 2001). (**F**, **G**) ∼12 hr old wt or *runt* mutant embryos, fixed and stained with α-Wg antibody; Wg sources in the visceral mesoderm are indicated by arrows. **DOI:** http://dx.doi.org/10.7554/eLife.09073.015

Staining *runt* mutant embryos with α-Labial antibody, we discovered that these mutants lack the middle midgut constriction, the signature phenotype found in all mutants with defective endoderm induction (including *wg* and *pygo*; Bienz, 1994; Thompson et al., 2002). Furthermore, staining is significantly weaker than in the wt, and exhibits no gradient (Figure 6C,D), mimicking the phenotype seen in *wg* mutants (Bienz, 1994; Thompson et al., 2002) (although Wg is expressed in *runt* mutants; Figure 6—figure supplement 1). Thus, *runt* is required for endoderm induction, likely for the Wg response of *labial*.

Next, we mutated the two Runt binding sites in the *labial* enhancer (*lab550R*), to test their function in transgenic lines with single copies of chromosomally integrated LacZ reporters. *lab550* produces a gradient of LacZ staining (Figure 6E) recapitulating endogenous Labial expression (Tremml and Bienz, 1992) (Figure 6C). This LacZ staining is much reduced in *runt* mutants, with no discernible gradient (Figure 6F), as in *wg* mutants. Similarly, *lab550R* mediates relatively weak and even LacZ expression, limited to a narrow band of cells (Figure 6G), which phenocopies the LacZ pattern from an enhancer with mutant dTCF binding sites (*lab550L*; Figure 6H). Importantly, *lab550* is strongly activated posteriorly if Runt is expressed throughout the endoderm (Figure 6I), while neither *lab500R* nor *lab550L* are Runt-responsive (Figure 6J,K). Thus, Runt acts through *lab550*, apparently cooperating with dTCF to render *lab550* signal-responsive. Likewise, the Runt binding sites in the *Ubx* midgut enhancer are also functional targets of Runt (Figure 6—figure supplement 1), implicating Runt in the Wg-dependent indirect autoregulation of *Ubx* at the top of the inductive cascade (Figure 6B).

### ChiLS associates with Groucho/TLE

**Figure 7.**
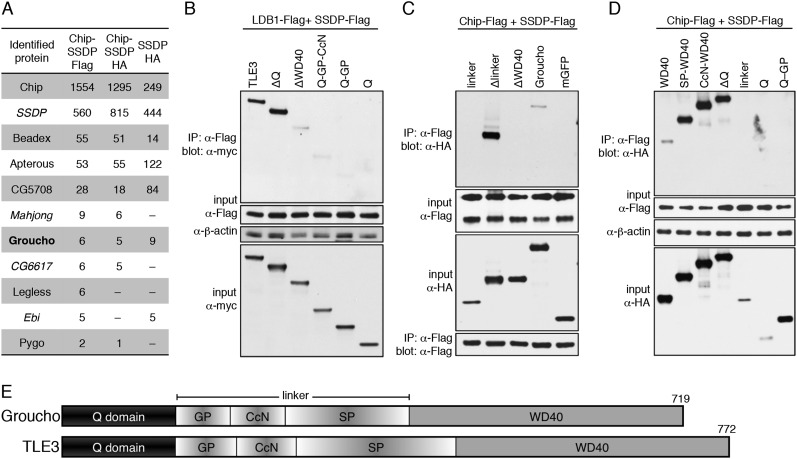
Groucho binds to ChiLS. (**A**) Top proteins associated with >2 baits (as indicated in table) in S2 cells, in addition to Legless and Pygo (unweighted spectral counts as in Figure 2A). (**B**–**D**) Western blots as in Figure 5C, showing coIP between co-expressed wt and truncated proteins as indicated above panels. (**E**) Cartoon of Groucho and TLE3, with domains indicated (GP, CcN, SP, semi-conserved elements within linker). **DOI:** http://dx.doi.org/10.7554/eLife.09073.016

Recall that neither Pygo nor ChiLS bind to DNA, nor to TCFs, raising the question how these proteins are recruited to TCF enhancers. To answer this, we used the same approach as described (Figure 2), to identify new ChiLS-interacting factors by mass spectrometry. Using SSDP as bait, we found Chip as the top hit (Figure 7A), confirming efficient complex formation between the two proteins (van Meyel et al., 2003; Xu et al., 2007b), comparable to that in the other two samples with co-overexpressed Chip and SSDP.

Looking for hits with similar spectral counts in all three coIPs provided a stringent criterion that eliminated most hits (many of which were false positives as they scored highly in the Flag but not HA IPs). The resulting overlap list contains Beadex, Apterous and CG5708 as the top hits, followed by Groucho (Figure 7A), the only new hit identified with high confidence. Additionally, two LisH domain-containing proteins (Ebi and CG6617) were identified with lower confidence, as well as Pygo and Legless (Figure 7A) whose low spectral counts may simply reflect low abundance (and S2 cells express neither Runt nor Lozenge).

CoIPs confirmed the interaction between ChiLS and Groucho or TLE3, and revealed that the WD40 domain is both necessary and sufficient for association, while the Q domain and linker are dispensable (Figure 7B–E). Even though the minimal WD40 domain coIPs only weakly with ChiLS, the same is true for full-length Groucho, while Δlinker interacts very strongly (Figure 7C), suggesting that the linker attenuates the interaction between Groucho/Tranducin-like enhancer protein (TLE) and ChiLS, consistent with the known regulatory role of this region (controlled by phosphorylation; Turki-Judeh and Courey, 2012). Groucho's binding to Chip neither requires its LID, nor SSDP (M. F., unpublished).

The WD40 propeller domain binds two classes of motifs in enhancer-binding proteins (WRPW/Y or en1, FxIxxIL) that ‘plug’ its pore (Jennings et al., 2006), but there are no convincing matches to either of these in ChiLS. Its interaction with WD40 may thus be mediated by a degenerate eh1-like or unknown motif, as found in other *bona fide* Groucho-binding proteins (Flores-Saaib et al., 2001; Turki-Judeh and Courey, 2012), or ChiLS may even bind to an alternative WD40 surface (for a precedent, see He et al., 2013). Crucially, since Groucho/TLE binds to TCF via its Q domain (Mieszczanek et al., 2008; Chodaparambil et al., 2014), it can thus function as an adaptor between ChiLS and TCF. We note that the WD40 domain also binds to the WRPY motif at the C-terminus of RUNX proteins (Canon and Banerjee, 2000; Chuang et al., 2013).

### ChiLS affects Wg responses in imaginal discs

*chip* is required for the function of remote enhancers of the homeobox genes *cut* (a Notch and Wg target which patterns the wing margin), and of *Ubx* (Morcillo et al., 1996) which specifies the middle body region including the third leg and haltere (the dorsal appendage that substitutes for the hind wing in flies). Furthermore, *chip* is required for Apterous-dependent wing development (Milan and Cohen, 1999), and for the notum bristles which are specified by Pannier and Achaete/Scute in a Wg-dependent fashion (Garcia–Garcia et al., 1999; Ramain et al., 2000). *ssdp* mutant clones produce wing defects that phenocopy *pygo* mutant clones (van Meyel et al., 1999). Transcriptional profiling of *ssdp* mutant wing discs identified numerous negatively-regulated SSDP target genes linked to Apterous and Pannier binding sites (Bronstein et al., 2010). Strikingly, the top scoring *positively*-regulated SSDP target genes were linked to dTCF binding sites (although this is not mentioned in the text, but see Table S3 in Bronstein et al., 2010)—a strong indication that SSDP promotes primarily dTCF-dependent transcription in this tissue.

Clonal analysis in wing discs confirmed that *chip* and *ssdp* control dTCF targets including *vestigial* and *wg*, similarly to *pygo* (Figure 8—figure supplement 1). In haltere discs, *ssdp* and *pygo* are required for high levels of Ubx expression, causing similar overgrowth phenotypes in halteres (Figure 8—figure supplement 2). These similarities between the *ssdp* and *pygo* mutant defects in the primordia for the dorsal appendages implicate both genes in the control of Wg-dependent master-regulatory genes.

### A pioneer-like function of ChiLS in the early embryo

*chip* has a pioneer-like role in the early embryo, when zygotic transcription starts at the maternal-zygotic transition, enabling expression of segmentation genes along the antero-posterior axis (Morcillo et al., 1997). We confirmed that embryos without maternal and zygotic Chip (*chip* germ-line clones, glcs) do not develop, except for rare escapers which show severe expression defects of segmentation genes, including *even-skipped* (*eve*) (Morcillo et al., 1997) (J. M., unpublished). Eve is a master-regulatory homeobox gene expressed in stripes along the embryonic antero-posterior axis where it activates numerous genes including *wg* (Kobayashi et al., 2001) and *Ubx* (Tremml and Bienz, 1989; Muller and Bienz, 1992).

**Figure 8.**
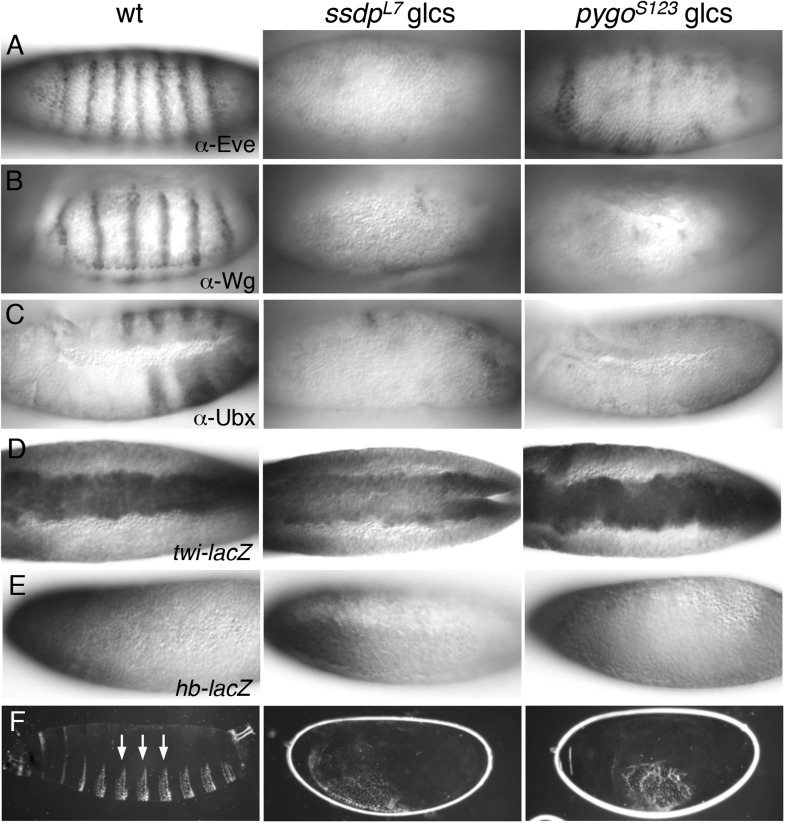
*ssdp* and *pygo* mutants show similar early embryonic defects. (**A**–**E**) 3–5 hr old wt and mutant glc embryos as indicated above panels, stained with antibodies as indicated in panels. (**F**) Larval cuticles of wt and mutant glc embryos; denticle belts (arrows) signify lack of Wg signaling. **DOI:** http://dx.doi.org/10.7554/eLife.09073.017

Nothing is known about *ssdp* function in embryos. Antibody staining of *ssdp* glcs revealed only residual expression of Eve, Wg and Ubx (Figure 8A–C). In contrast, *twi*-*LacZ* (recapitulating endogenous *twist*, a mesoderm-specifying bHLH gene activated in the ventral-most zone; Thisse et al., 1991) appears normal in *ssdp* glcs (Figure 8D). Likewise, *hb-LacZ* (recapitulating expression of endogenous *hunchback*, a gene specifying anterior development; Struhl et al., 1992) is normal in these mutants (Figure 8E). These *ssdp* defects phenocopy those of *chip* glcs (Morcillo et al., 1997), and they indicate that ChiLS is required for gene activity along the antero-posterior but not the dorso-ventral axis. An important corollary is that Wg is never expressed in embryos lacking ChiLS. In support of this, the cuticles of the rare *chip* glc escapers show a lawn of ventral denticles (Morcillo et al., 1997), as do *ssdp* glcs (Figure 8F) and *pygo* glcs (Parker et al., 2002; Thompson et al., 2002)—the hallmark of Wg signaling failure (Cadigan and Nusse, 1997).

**Figure 8—figure supplement 1.**
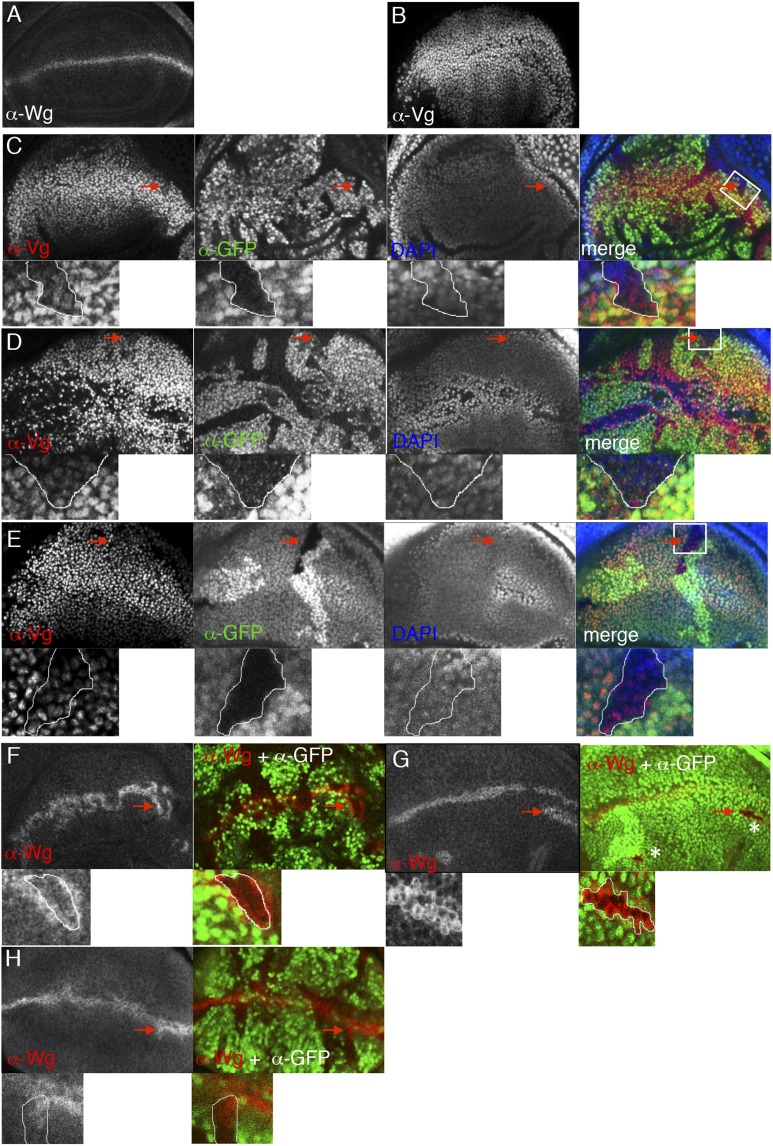
ChiLS is required for Wg and Notch responses in wing discs. (**A**, **B**) Single confocal sections through third larval instar wing discs, fixed and stained with α-Wg or α-Vg antibody, as indicated in panels. *wg* is activated by Notch signaling along the prospective anterior margin of the third instar disc, while *vg* is expressed in a Wg-dependent fashion in a broad zone straddling Wg expression along the prospective margin and sustains proliferation in the prospective wing blade (Couso et al., 1995; Rulifson and Blair, 1995; Kim et al., 1996; Klein and Arias, 1999; Micchelli et al., 1997). (**C**–**E**) Single confocal sections of wing discs as in (**A**, **B**), bearing (**C**) *pygo*^*S123*^, (**D**) *ssdp*^*L7*^ or (**E**) *chip*^*e55*^ mutant clones, stained with α-Vestigial (Vg) (red in merge); clones are marked by absence of GFP (green in merge); all discs were counterstained with DAPI (blue in merge), to label the nuclei (as internal control for the focal plane). Reduction of Vg expression within mutant clones of all three genes is best detectable in the ventral compartment (without Apterous expression) at a distance from the Wg source, as shown in high-magnification insets below panels (from marked squares, marked in merges; clones demarcated by white lines), as previously reported for *dTCF* mutant clones that are unable to respond to Wg signaling (Schweizer et al., 2003). (**F**–**H**) Single confocal sections of wing discs as in (**C**–**E**), bearing (**F**) *ssdp*^*L7*^, (**G**) *chip*^*e55*^ or (**H**) *pygo*^*S123*^ mutant clones, stained with α-Wg (red in merge); clones are marked by absence of GFP (green in merge), and discs were counterstained with DAPI; insets below panels are high-magnification views of selected clones (marked by squares in merges) revealing ectopic activation of Wg along the edge of the clone (signifying activation by Notch signaling derived from adjacent wt cells, combined with loss of Wg-mediated repression within the clone; Rulifson and Blair, 1995; Rulifson et al., 1996). Note also that, in the dorsal disc (the Apterous territory), *chip*^*e55*^ mutant clones barely survive (indicated by asterisks in the merge in **G**), compared to *ssdp*^*L7*^ mutant clones that appear to grow normally, like *pygo*^*S123*^ mutant clones; however, in the absence of Apterous, i.e. in the ventral compartment, *chip*^*e55*^ mutant clones grow normally (see clone marked by arrow in **E**). Collectively, our clonal analysis indicates that ChiLS is required for Wg and Notch responses in the prospective wing blade and margin, consistent with previous results (Morcillo et al., 1996; Morcillo et al., 1997; van Meyel et al., 2003) similarly to Pygo (Belenkaya et al., 2002; Parker et al., 2002; Thompson et al., 2002), although Chip exhibits a far more stringent requirement for proliferation in the Apterous territory than either SSDP or Pygo. **DOI:** http://dx.doi.org/10.7554/eLife.09073.018

**Figure 8—figure supplement 2.**
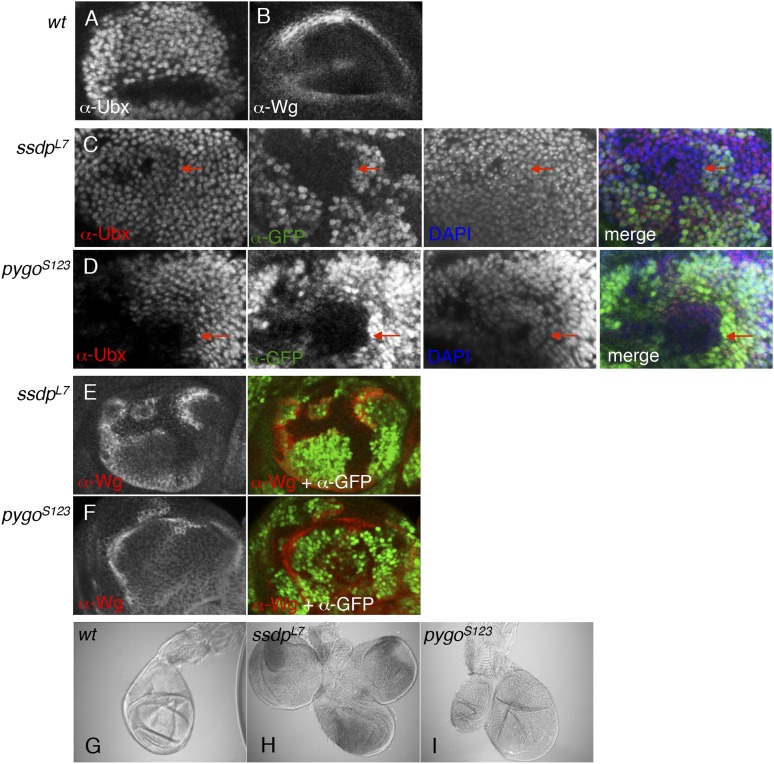
ChiLS is required for Ubx and Wg expression in haltere discs. (**A**, **B**) Single confocal sections through third larval instar haltere discs, fixed and stained with α-Ubx or α-Wg antibody, as indicated in panels. *Ubx* specifies haltere development by attenuating *vg* and proliferation in haltere discs, partly through interaction with Wg (Prasad et al., 2003). (**C**, **D**) Single confocal sections of haltere discs as in (**A**, **B**), bearing *pygo*^*S123*^ or *ssdp*^*L7*^ mutant clones as indicated in panels, stained with α-Ubx antibody (red in merge); clones are marked by absence of GFP (green in merge); all discs were counterstained with DAPI (blue in merge), to label the nuclei (as internal control for the focal plane). Note the reduction of Ubx expression in clones marked by red arrows, which results in slight overproliferation and outgrowth of clone (as judged by the bulging out from the focal plane of the mutant epithelium). (**E**, **F**) Single confocal sections of clone-bearing haltere discs as in (**C**, **D**), stained with α-Wg antibody (red in merge); clones are marked by absence of GFP (green in merge), and counterstained with DAPI. Note the ectopic expression of Wg along the edges within the mutant clones, similarly to the clones shown in Figure 8—figure supplement 1 (**F**–**H**), likely as a result of the same Notch-stimulatory and Wg autoinhibitory and inputs. (**G**–**I**) Patterning defects in halteres, reflecting overgrowth of *pygo*^*S123*^ or *ssdp*^*L7*^ mutant clones due to reduced Ubx and ectopic Wg expression in the disc. **DOI:** http://dx.doi.org/10.7554/eLife.09073.019

We also examined glcs bearing a *pygo* null allele (Thompson et al., 2002), which revealed considerable defects in Eve and Ubx expression, and almost complete absence of Wg (Figure 8A–C), as previously reported for a different null *pygo* allele (Parker et al., 2002). These Wg-independent defects are not detectable with weaker *pygo* truncation alleles that merely lack the PHD finger (Kramps et al., 2002; Thompson et al., 2002), confirming that the (ChiLS-dependent) activation of segmentation genes along the antero-posterior axis resides in the NPF-containing N-terminus of Pygo.

## Discussion

**Figure 9.**
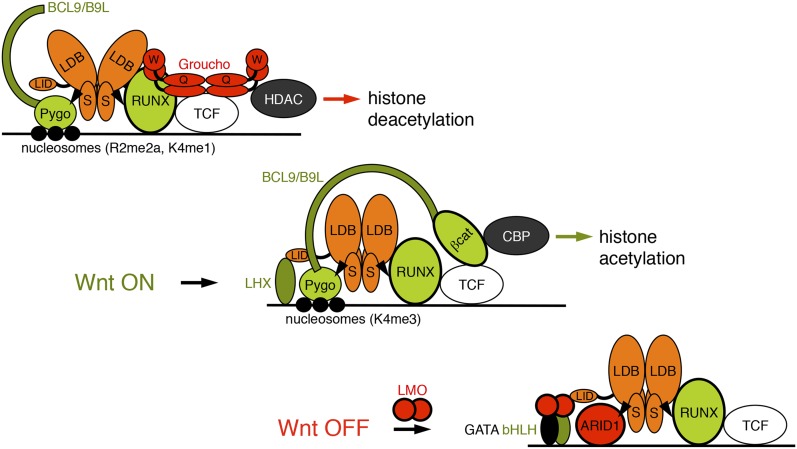
Model of the Wnt enhanceosome. Switching of the Wnt enhanceosome from OFF (*top*) to ON (*middle*), and towards re-repression (*bottom*), prefacing re-recruitment of Groucho/TLE; S, SSDP; Q, Q domain; W, WD40 domain; NPF-mediated interactions are indicated by arrowheads; green, positively-acting components; red, negatively-acting components; known oncogenes and tumor suppressors are circled in bold. The stoichiometry of ChiLS allows simultaneous interaction with NPF and LID-binding factors, although these may also displace each other, as indicated (except for RUNX, arbitrarily shown as ChiLS-associated throughout). Indicated conformational changes are entirely hypothetical. **DOI:** http://dx.doi.org/10.7554/eLife.09073.020

Our discovery of ChiLS as the NPF ligand of Pygo proteins led us to define the core components of a multi-protein complex tethered to TCF enhancers via Groucho/TLE and RUNX, and slated for subsequent Wnt responses by Pygo (Figure 9). ChiLS also contacts additional enhancer-binding proteins via its LID, including lineage-specific and other signal-responsive factors (Bronstein and Segal, 2011), thereby integrating multiple position-specific inputs into TCF enhancers, which provides a molecular explanation for the context-dependence of TCF/LEF. We shall refer to this complex as the Wnt enhanceosome since it shares fundamental features with the paradigmatic interferon β-responsive enhanceosome (Panne et al., 2007). Its components are conserved in placozoa, arguably the most primitive animals without axis and tissues with only a handful of signaling pathways including Wnt, Notch and TGF-β/SMAD (Ringrose et al., 2013), suggesting that the Wnt enhanceosome emerged as the ur-module integrating signal-responses.

Other proteins have been reported to interact with the Pygo N-terminus (Carrera et al., 2008; Wright and Tjian, 2009; Chen et al., 2010), but none of these recognize NPF. We note that this N-terminus is composed of low-complexity (intrinsically disordered) sequences that are prone to non-specific binding.

### Recognition of NPF by ChiLS

NPF is a versatile endocytosis motif that binds to structurally distinct domains (Mahadev et al., 2007), including eps15 homology (EH) domains in epsin15 homology domain (EHD) proteins (Kieken et al., 2010). Indeed, we consistently identified EHDs in lysate-based pull-downs with triple-NPF baits (Figure 2—figure supplement 1D). EHDs are predominantly cytoplasmic, and do not interact with nuclear Pygo upon co-expression (M. G., unpublished), nor are any of the *Drosophila* EHDs required for Wg signaling in S2 cells (Stadeli and Basler, 2005). ChiLS is the first nuclear NPF-binding factor.

NPF binding to ChiLS appears to depend on the same residues as NPF binding to EHD domains (de Beer et al., 2000), that is, on the aromatic residue at +2 (Figure 4), the invariant P at +1, N (or G) at 0 and NPF-adjacent residues (Figure 5A), including negative charges at +3 and +4 (whereby a positive charge at +3 abolishes binding to EHD; Kieken et al., 2010). Indeed, an intramolecular interaction between the +3 side-chain and that of N predisposes NPF to adopt a type 1 β-turn conformation, which increases its affinity to the EHD pocket, while the −1 residue undergoes an intermolecular interaction with this pocket (de Beer et al., 2000). ChiLS also shows a preference for small residues at −1 and −2, similarly to N-terminal EHDs (Paoluzi et al., 1998) although RUNX seems to differ at −1 and −2 from Pygo and MACC1 (F/L A/E/D vs S A, respectively).

### RUNX as the keystone of the Wnt enhanceosome?

Groucho/TLE is recruited to TCF via its Q domain, which tetramerizes (Chen et al., 1998; Chodaparambil et al., 2014). Intriguingly, the short segment that links two Q domain dimers into a tetramer (Chodaparambil et al., 2014) is deleted in a dTCF-specific *groucho* allele that abolishes dTCF binding and Wg responses (Mieszczanek et al., 2008), suggesting that TCF may normally bind to a Groucho/TLE tetramer.

Groucho/TLE uses its second domain, the WD40 propeller, to bind to other enhancer-binding proteins on Wnt-responsive enhancers (Turki-Judeh and Courey, 2012), most notably to the C-terminal WRPY motif of RUNX proteins (common partners of TCFs in Wnt-responsive enhancers; Figure 6A). This interaction can occur simultaneously with the WD40-dependent binding to ChiLS (Figure 7F), given the tetramer structure of Groucho/TLE. In turn, RUNX uses its DNA-binding Runt domain to interact with HMG domains of TCFs (Kahler and Westendorf, 2003; Ito et al., 2008), and to recruit ChiLS (Figure 5—figure supplement 3). RUNX thus appears to be the keystone of the Wnt enhanceosome since it binds to the enhancer directly while undergoing simultaneous interactions with Groucho/TLE (through its C-terminal WRPY motif), TCF and ChiLS (though its Runt domain).

In line with this, Runt has pioneering functions in the early *Drosophila* embryo, shortly after the onset of zygotic transcription (Canon and Banerjee, 2000), and in the naïve endoderm (Figure 6) as soon as this germlayer is formed, in each case prior to the first Wg signaling events. RUNX paralogs also have pioneer-like functions in specifying cell lineages, that is, definitive hematopoiesis in flies and mammals (Chuang et al., 2013).

### Switching between ON and OFF states

Our model predicts that ChiLS, once tethered to the enhanceosome core complex, recruits Pygo via NPF to prime the enhancer for Wnt responses (Figure 9). Given the dimer-tetramer architecture of ChiLS (Figure 3C), its binding to Pygo can occur simultaneously to its NPF-dependent binding to RUNX. In turn, tethering Pygo to the Wnt enhanceosome may require Pygo's binding to methylated histone H3 tail (Fiedler et al., 2008; Miller et al., 2013), similarly to Groucho/TLE whose tethering to enhancers depends on binding to hypoacetylated histone H3 and H4 tails (Sekiya and Zaret, 2007). Interestingly, Pygo's histone binding requires at least one methyl group at K4 (Fiedler et al., 2008; Miller et al., 2013)—the hallmark of poised enhancers (Kharchenko et al., 2011). Indeed, *Drosophila* Pygo is highly unorthodox due to an architectural change in its histone-binding surface that allows it to recognize asymmetrically di-methylated arginine 2 (Miller et al., 2013)—a hallmark of silent chromatin (Kirmizis et al., 2007). Thus, the rare unorthodox Pygo proteins (Miller et al., 2013) may recognize silent enhancers even earlier, long before their activation, consistent with the early embryonic function of Pygo, prior to Wg signaling (Figure 8).

Overcoming the OFF state imposed on the enhancer by Groucho/TLE (Mieszczanek et al., 2008) involves Pygo-dependent capturing of β-catenin/Armadillo, which recruits various transcriptional co-activators to its C-terminus (Mosimann et al., 2009). Although these include CREB-binding protein (CBP), a histone acetyl transferase, its tethering to TCF enhancers is likely to co-depend on CRE-binding factors (CREB, c-Fos) and SMAD (Bienz, 1997; Waltzer and Bienz, 1999) which synergize with Armadillo to activate these enhancers (Bienz, 1997; Waltzer and Bienz, 1999)—similarly to the interferon-β enhanceosome where CBP recruitment also co-depends on multiple enhancer-binding proteins (Panne et al., 2007). The ensuing acetylation of the Wnt enhancer chromatin could promote the eviction of Groucho/TLE whose chromatin anchoring is blocked by acetylation of histone H3 and H4 tails (Chodaparambil et al., 2014), thus initiating the ON state.

Osa antagonizes Wg responses throughout development, and represses *UbxB* through its CRE (Collins and Treisman, 2000), which also mediates repression in response to high Wg signaling (Yu et al., 1998) (Figure 6B). Osa could therefore terminate enhancer activity, by displacing HAT-recruiting enhancer-binding proteins such as CREB and c-Fos from CREs and by cooperating with repressive enhancer-binding proteins such as Brinker (a Groucho-recruiting repressor that displaces SMAD from *UbxB*; Saller and Bienz, 2001; Saller et al., 2002) to re-recruit Groucho/TLE to the enhancer, thereby re-establishing its OFF state (Figure 9). Notably, Osa binds Chip, to repress various Wg and ChiLS targets including *achaete-scute* and *dLMO* (Heitzler et al., 2003; Milan et al., 2004).

Therefore, ChiLS is not only a coincidence detector of multiple enhancer-binding proteins and NPF proteins, but also a switch module that exchanges positively- and negatively-acting enhancer-binding proteins (through LID) and NPF factors, to confer signal-induced activation, or re-repression (Figure 9). Its stoichiometry and modularity renders it ideally suited to both tasks. We note that the interferon-β enhanceosome does not contain a similar integrating module (Panne et al., 2007), perhaps because it is dedicated to a single signaling input.

### ChiLS is a pioneer-like co-regulator

ChiLS is essential for activation of master-regulatory genes in the early embryo (Morcillo et al., 1997) (Figure 8), similarly to DNA-binding pioneer factors such as Zelda (in the *Drosophila* embryo) or FoxA (in the mammalian endoderm) which render enhancers accessible to enhancer-binding proteins (Zaret and Carroll, 2011). Moreover, ChiLS maintains HOX gene expression throughout development (Figure 5; Figure 8—figure supplement 2), enabling Wg to sustain HOX autoregulation, a mechanism commonly observed to ensure coordinate expression of HOX genes in groups of cells (Bienz, 1994).

Another hallmark of pioneer factors is that they initiate communication with the basal transcription machinery associated with the promoter. Chip is thought to facilitate enhancer-promoter communication (Morcillo et al., 1997), possibly by bridging enhancers and promoters through self-association (Cross et al., 2010) (Figure 3). Indeed, Ldb1 occupies both remote enhancers and transcription start sites (e.g., of globin genes and *c-Myb*; Love et al., 2014), likely looping enhancers to the basal transcription machinery at promoters (Deng et al., 2012; Stadhouders et al., 2012) which requires self-association (Krivega et al., 2014), but possibly also other factors (such as cohesin, or mediator; Levine et al., 2014).

We note that the chromatin association of Ldb1 has typically been studied in erythroid progenitors or differentiated erythroid cells (Love et al., 2014), following activation of erythoid-specific genes (Stadhouders et al., 2012). It would be interesting (if technically challenging) to examine primitive cells, to determine whether ChiLS is associated exclusively with poised enhancers prior to cell specification or signal responses.

### Enhanceosome switching from Wnt to Notch responses

Previous genetic analysis in *Drosophila* has linked *chip* predominantly to Notch-regulated processes (Bronstein and Segal, 2011). Likewise, *groucho* was initially thought to be dedicated to repression downstream of Notch (Preiss et al., 1988), before its role in antagonizing TCF and Wnt responses emerged (Roose and Clevers, 1999). Moreover, Lozenge facilitates Notch responses in the developing eye, and in hematocytes (Canon and Banerjee, 2000; Terriente-Felix et al., 2013). Indeed, the first links between Groucho/TLE, RUNX and nuclear Wnt components came from physical interactions (Roose and Clevers, 1999), as in the case of ChiLS (Figure 2). Our work indicates that these nuclear Notch signaling components constitute the Wnt enhanceosome. Although our most compelling evidence for this notion is based on physical interactions, the genetic evidence from *Drosophila* is consistent with a role of ChiLS in Wg responses (Bronstein et al., 2010) (Figure 6; Figure 8—figure supplements 1, 2). Indeed, mouse *Ldb1* has been implicated in Wnt-related processes, based on phenotypic analysis of *Ldb1* knock-out embryos and tissues (Mukhopadhyay et al., 2003; Mylona et al., 2013). Notably, *Ldb1 *has wide-spread roles in various murine stem cell compartments that are controlled by Wnt signaling (Xu et al., 2007a; Dey-Guha et al., 2009; Li et al., 2011; Salmans et al., 2014).

An interesting corollary is that the Wnt enhanceosome may be switchable to Notch-responsive, by NPF factor exchange and/or LMO-mediated enhancer-binding protein exchange at ChiLS (Figure 9). Hairy/Enhancer-of-split (HES) repressors could be pivotal for this switch (Delidakis et al., 2014): these bHLH factors are universally induced by Notch signaling, and they bind to ChiLS enhancers to re-recruit Groucho/TLE via their WRPW motifs (Turki-Judeh and Courey, 2012). HES repressors may thus be capable of re-establishing the OFF state on Wnt enhancers in response to Notch.

Notably, restoring a high histone-binding affinity in *Drosophila* Pygo by reversing the architectural change in its histone-binding surface towards human renders it hyperactive towards both Wg and Notch targets (Miller et al., 2013) even though *pygo* is not normally required for Notch responses in flies. Humanized Pygo may thus resist the Notch-mediated shut-down of the Wnt enhanceosome, owing to its elevated histone affinity that boosts its enhancer tethering, which could delay its eviction from the enhanceosome by repressive NPF factors. The apparent Notch-responsiveness of the Wnt enhanceosome supports our notion that orthodox Pygo proteins (as found in most animals and humans) confer both Wnt and Notch responses (Miller et al., 2013).

### Links to human cancer

Previous genetic studies have shown that the components of the Wnt enhanceosome (e.g., TCF, RUNX, ChiLS and LHX) have pivotal roles in stem cell compartments, as already mentioned (see also Folgueras et al., 2013; Lien and Fuchs, 2014), suggesting a universal function of this enhanceosome in stem cells. It is therefore hardly surprising that its dysregulation, that is, by hyperactive β-catenin, is a root cause of cancer, most notably colorectal cancer but also other epithelial cancers (Clevers, 2006). Indeed, genetic evidence implicates almost every one of its components (as inferred from the fly counterparts) in cancer: AML1 and RUNX3 are tumour suppressors whose inactivation is prevalent in myeloid and lymphocytic leukemias (Mangan and Speck, 2011), and in a wide range of solid tumors including colorectal cancer (Chuang et al., 2013), respectively. Likewise, ARID1A is a wide-spread tumor suppressor frequently inactivated in various epithelial cancers (Wu and Roberts, 2013). Furthermore, many T-cell acute leukemias can be attributed to inappropriate expression of LMO2 (Rabbitts, 1998). Intriguingly, AML1 and ARID1A behave as haplo-insufficient tumor suppressors, consistent with the notion that these factors compete with activating NPF factors such as Pygo2, RUNX2 and possibly MACC1 (predictive of metastatic colorectal cancer; Stein et al., 2009) for binding to ChiLS, which will be interesting to test in future. The case is compelling that the functional integrity of the Wnt enhanceosome is crucial for the avoidance of cancer.

## Materials and methods

### Protein purification

6xHis-MBP-Chip_205-436,_ 6xHis-MBP-LDB1_56-285_ and 6xHis-Lip-SSDP_1-92_ were co-expressed with a bi-cistronic expression vector (including N-terminal Tobacco Etch virus (TEV) protease sites for removal of tags) in *E. coli* BL21-CodonPlus(DE3)-RIL cells (Stratagene, La Jolla, California, United States) and purified by Ni-NTA resin and size exclusion chromatography, as described (Fiedler et al., 2008). 6xHis-Lip-tagged NPF-containing fragments (Figures 4, and 5) were purified similarly, after labeling in minimal media for NMR (Miller et al., 2010, 2013).

### Mass spectrometry

*Drosophila* S2 cells were grown in Lonza serum-free medium and transfected with bait plasmids (Figure 2—figure supplement 1A) using Fugene HD and subsequently grown under continuous selection with 5 μg ml^−1^ puromycin. For tandem-affinity purification of Pygo-associated proteins, ∼2 × 10^9^ S2 cells (grown as suspension culture) or twenty 175 cm^2^ flasks of subconfluent HEK293T cells stably transfected with Pygo baits were used for each experiment. Cells were lysed in 30 ml lysis buffer (12.5 mM Tris–HCl pH 7.4, 6.25% glycerol, 125 mM NaCl, 0.625 mM EDTA, 3.1 mM NaF, 1.25 mM Na_3_PO_4_, 0.125% Triton-X-100), and sonicated 4 × 10 s at 50% intensity with a Branson 250 Sonifier. Cell lysates were cleared by centrifugation for 20 min at 15,000 rpm, and incubated (while rotating) for 1 hr with α-Flag affinity resin (Sigma) in the cold room. Immunoprecipitates were washed 4x with 1 ml lysis buffer, and subsequently eluted with 4 consecutive 500 μl elutions of lysis buffer supplemented with 200 µg ml^−1^ 3xFlag-Peptide (Sigma). Eluates were subjected to α-Strep pull-down with 20 µl packed volume of StrepTactin (IBA Lifesciences). Beads were then washed 3x with 2 ml lysis buffer, and subsequently boiled in 50 µl 2× LDS sample buffer. Proteins were resolved on 4–12% Bis-Tris SDS-polyacrylamide gels. These were stained with Imperial Protein Stain (Thermoscientific), and gel lanes were cut into 1–2 mm slices for in situ digestion with trypsin. The analytical column outlet was directly interfaced via a modified nano-flow electrospray ionisation source, with a hybrid linear quadrupole ion trap mass spectrometer (Orbitrap LTQ XL, ThermoScientific, San Jose, United States). LC-MS/MS data were searched against a protein database (UniProt KB) with the Mascot search engine program (Matrix Science, UK) (Perkins et al., 1999). MS/MS data were validated using the Scaffold programme (Proteome Software Inc., United States).

### SEC-MALS

100 μl SSDP, 6xHis-MBP-Chip–6xHis-Lip-SSDP or 6xHis-MBP-LDB1–6xHis-Lip-SSDP samples were resolved on a Superdex S-200 or Superose 6 HR 10/300 analytical gel filtration column (GE Healthcare) at 0.5 ml min^−1^ in 25 mM phosphate buffer, 150 mM NaCl, pH 6.7 before light scattering and concentration determination using refractive index (RI) or UV absorbance in a standard SEC-MALS configuration (containing a Wyatt Heleos II 18 angle light scattering instrument coupled to a Wyatt Optilab rEX online RI detector). Protein concentration was determined from the excess differential refractive index based on 0.186 RI increment for 1 g ml^−1^ protein solution. Concentrations and observed scattered intensities at each point in the chromatograms were used to calculate absolute molecular mass from the intercept of the Debye plot, using Zimm's model as implemented in Wyatt's ASTRA software. The stoichiometries of ChiLS indicated by the model-free RI measurements (using dn/dc 0.186 for 1g ml^−1^ protein) were further confirmed by using appropriate UV extinction coefficients and UV absorbance as the concentration measurement, which produced essentially identical masses to those from RI. They were independent of protein concentration (in the range of 0.1–10 mg ml^−1^).

### NMR spectroscopy

[^1^H,^15^N]fast-HSQC spectra of ^15^N-labeled proteins in 25 mM phosphate buffer, 150 mM NaCl were recorded with 600 MHz ^1^H frequency (at 25°C), and ^13^C/^15^N double-labeled samples were used for backbone resonance assignments. Datasets were acquired, processed and analyzed as described (Miller et al., 2013).

### Plasmids and cell-based assays

The following plasmids were recloned in pCMVtag2b, for transfecting HEK293T cells (with PEI at a ratio of 1:3.5, DNA:PEI): Chip (Morcillo et al., 1997), SSDP (van Meyel et al., 1999), Groucho (Jennings et al., 2006), LDB1 (from Luc Sabourin), AML1, Runx2-P1, Runx3-P1 (from Anna Kilbey and Karen Blyth), monomeric GFP (mGFP, from John James). HA-Pygo, HA-hPygo2 (Thompson et al., 2002) and 6xMyc-TLE3 (Hanson et al., 2012) were also used. Internal deletions were generated by standard procedures in the same vectors, and verified by sequencing. Cell culture, lysate preparation and coIPs were done essentially as described (Thompson et al., 2002). The following antibodies and antibody-coupled resins were used: α-LDB1 (Epitomics); α-β-actin (Abcam); α-Flag M2, α-HA (Sigma).

### Fly assays

All *Drosophila* strains used are described in Flybase. The following new transgenic lines bearing mutant enhancers were generated: *UbxBR* was derived from mutating *UbxB* (in a *ry*^*+*^ vector; Thuringer et al., 1993), and 2 independent transformant lines were isolated by standard procedures. Likewise, *lab550R* and *lab550L* were derived from mutating *lab550* (in a *ry*^*+*^ vector; Tremml and Bienz, 1992), and 5 independent transformat lines were isolated. The following mutations were made (numbers refer to binding site number, from 5′ to 3′): *UbxB Runt1*, AACCTCG > CTCTAGA; *UbxB Runt2*, TCTGGTA > CTCTAGA; *lab550 Runt1*, TTTGGTT > AAGATCT; *lab550 Runt2*, TGTGGTC > AAGATCT; *lab550 dTCF1*, TTACAAA > GCCGGCA; *lab550 dTCF2*, CATCAAT > GGGCCCT; *lab550 dTCF3*, CATCAAC > CTCGAGC; *lab550 dTCF4*, GTTGATG > GaGTACTG (‘a’ denotes a one-base insertion in this mutant dTCF binding site); only *dTCF1* and *dTCF2* binding sites are shown in Figure 6A (depicting the 5′ portion of the *lab550* enhancer) while *dTCF3* and *dTCF4* are near the 3′ end of *lab550*. *ssdp* and *pygo* mutant wing disc clones were generated with *vg.GAL4 UAS.flp* as described (Vegh and Basler, 2003; de la Roche and Bienz, 2007; Fiedler et al., 2008), but *hs.flp* was used for *chip* mutant clones (which were generated by heat-shocking late second or early third instar larvae for 30 min at 37°C). The GAL4 drivers used for overexpressing Runt (Tracey et al., 2000) are described in Flybase (*24B.GAL4*, for mesodermal expression; *48Y.GAL4*, for endodermal expression). Paraformaldehyde-fixed embryos were stained with α-Labial (Tremml and Bienz, 1992), α-Eve (Azpiazu et al., 1996), α-Ubx, α-Wg (Developmental Studies Hybridoma Bank), α-β-galactosidase (Promega) as described (Thompson et al., 2002). DIC optics were used for imaging embryos on a Zeiss Axiophot. Paraformaldehyde-fixed imaginal discs were stained with α-Vg (Kim et al., 1996), α-Ubx, α-Wg (Developmental Studies Hybridoma Bank), rabbit or mouse α-GFP (Sigma) as described (Thompson et al., 2002; Fiedler et al., 2008). All discs were counterstained with DAPI, to control for the focal plane, and single confocal images were acquired at identical settings with a Zeiss Confocal Microscope.
